# Characterization of the Cellular Microenvironment and Novel Specific Biomarkers in Pterygia Using RNA Sequencing

**DOI:** 10.3389/fmed.2021.714458

**Published:** 2022-01-31

**Authors:** Julian Wolf, Rozina Ida Hajdu, Stefaniya Boneva, Anja Schlecht, Thabo Lapp, Katrin Wacker, Hansjürgen Agostini, Thomas Reinhard, Claudia Auw-Hädrich, Günther Schlunck, Clemens Lange

**Affiliations:** ^1^Eye Center, Medical Center, Faculty of Medicine, University of Freiburg, Freiburg im Breisgau, Germany; ^2^Department of Ophthalmology, Semmelweis University, Budapest, Hungary; ^3^Institute of Anatomy and Cell Biology, Wuerzburg University, Wuerzburg, Germany; ^4^Ophtha-Lab, Department of Ophthalmology, St. Franziskus Hospital, Münster, Germany

**Keywords:** conjunctival pterygium, RNA sequencing, FFPE, xCell, cellular microenvironment

## Abstract

With a worldwide prevalence of ~12%, pterygium is a common degenerative and environmentally triggered ocular surface disorder characterized by wing-shaped growth of conjunctival tissue onto the cornea that can lead to blindness if left untreated. This study characterizes the transcriptional profile and the cellular microenvironment of conjunctival pterygia and identifies novel pterygia-specific biomarkers. Formalin-fixed and paraffin-embedded pterygia as well as healthy conjunctival specimens were analyzed using MACE RNA sequencing (*n* = 8 each) and immunohistochemistry (pterygia *n* = 7, control *n* = 3). According to the bioinformatic cell type enrichment analysis using xCell, the cellular microenvironment of pterygia was characterized by an enrichment of myofibroblasts, T-lymphocytes and various antigen-presenting cells, including dendritic cells and macrophages. Differentially expressed genes that were increased in pterygia compared to control tissue were mainly involved in autophagy (including *DCN, TMBIM6*), cellular response to stress (including *TPT1, DDX5*) as well as fibroblast proliferation and epithelial to mesenchymal transition (including *CTNNB1, TGFBR1, and FN1*). Immunohistochemical analysis confirmed a significantly increased FN1 stromal immunoreactivity in pterygia when compared to control tissue. In addition, a variety of factors involved in apoptosis were significantly downregulated in pterygia, including *LCN2, CTSD*, and *NISCH*. Furthermore, 450 pterygia-specific biomarkers were identified by including transcriptional data of different ocular surface pathologies serving as controls (training group), which were then validated using transcriptional data of cultured human pterygium cells. Among the most pterygia-specific factors were transcripts such as *AHNAK, RTN4, TPT1, FSTL1*, and *SPARC*. Immunohistochemical validation of SPARC revealed a significantly increased stromal immunoreactivity in pterygia when compared to controls, most notably in vessels and intravascular vessel wall-adherent mononuclear cells. Taken together, the present study provides new insights into the cellular microenvironment and the transcriptional profile of pterygia, identifies new and specific biomarkers and in addition to fibrosis-related genes, uncovers autophagy, stress response and apoptosis modulation as pterygium-associated processes. These findings expand our understanding of the pathophysiology of pterygia, provide new diagnostic tools, and may enable new targeted therapeutic options for this common and sight-threatening ocular surface disease.

## Introduction

Pterygium is a common degenerative and environmentally triggered disease of the ocular surface characterized by wing-shaped growth of epithelial and fibrovascular conjunctival tissue on the cornea with a worldwide prevalence of ~12% (1). Visual impairment can occur as a result of induced astigmatism and involvement of the optical axis. Confirmed risk factors are increasing age, male gender and ultraviolet light exposure (1), which is supported by a direct correlation between proximity to the equator and prevalence (2). However, the underlying causes for the development of pterygia are not yet fully understood. In addition to ultraviolet radiation, several mechanisms are discussed that promote the development of pterygia, including epithelial mesenchymal transition, immunological and anti-apoptotic mechanisms, viral infections, angiogenic stimulation, and dysregulation of growth factors (2, 3). The current treatment is based on surgical removal and autologous conjunctival transplantation in combination with cytostatic and/or immunomodulatory therapy (4). Although adjuvant therapy has significantly reduced the recurrence rate compared to surgical resection alone, recurrence still occurs in about 5% of cases (4). To further improve the understanding of the pathogenesis as well as the treatment of the disease, a transcriptome analysis provides useful information about the underlying cellular and molecular mechanisms and about new potential diagnostic and therapeutic targets.

To date, a number of studies have used microarray technology to analyze the expression profile of pterygium samples (5–10), a method which is limited by technical issues, including limited probe coverage, inconsistent probe hybridization efficiency and its insensitivity to transcripts of low abundance (11, 12). RNA sequencing technology, in contrast, allows a more accurate and unbiased analysis of gene expression with less technical variation and a lower false positive rate and is additionally able to detect novel and rare transcripts that have previously been missed by conventional microarray technology (11, 12). So far, there are only a limited number of studies that have applied RNA sequencing on pterygia, including two studies using cultured pterygium cells (13, 14) and two recently published studies based on surgically removed pterygium tissue (15, 16). However, the aforementioned studies are limited by the use of postmortem control tissue (16) as well as by small sample sizes or by controls obtained from pterygium-affected eyes (15), so an influence of the disease as well as associated environmental factors on control tissue cannot be excluded.

The present study uses RNA sequencing to characterize the cellular microenvironment and the transcriptional profile of surgically removed pterygia compared to healthy conjunctival specimens, applies immunohistochemistry to validate key pterygium-associated factors and identifies pterygium-specific marker genes by including the transcriptional profiles of different ocular surface diseases. The results provide new insights into the pathways, molecular mechanisms and cell types involved in the pathogenesis of the disease, reveal new diagnostic markers and may lead to new options of targeted therapy for pterygia.

## Methods

### Patients

A total of 8 pterygium samples from 8 patients who underwent surgery at the Eye Center of the University of Freiburg between 2015 and 2018 were retrospectively included for transcriptome analysis. Eight healthy conjunctival specimens from 8 patients who underwent retinal detachment surgery but with no other history of ocular surface diseases served as controls. For immunohistochemistry, 7 pterygium samples and 3 healthy controls were analyzed (resection at our institution between 2013 and 2020). All methods were carried out in accordance with relevant guidelines and regulations and informed consent was obtained from all patients. To identify pterygium-specific markers, the transcriptional profiles of 26 ocular surface tumor specimens, among them conjunctival melanoma (*n* = 12), squamous cell carcinoma (*n* = 7) and papilloma (*n* = 7), were included, which were recently generated and published by our group using identical sequencing methods (17–19). Demographic data for these 26 patients are available in the corresponding publications (18, 19).

### Formalin Fixation and Paraffin Embedding

Formalin fixation and paraffin embedding (FFPE) of tissue samples was performed immediately after surgery according to routine protocols, as previously described (20, 21). Briefly, samples were fixed immediately after surgery in 4% formalin for 12 h, dehydrated in alcohol and processed for paraffin embedding. Histological diagnoses were made by two experienced ophthalmic pathologists.

### Immunohistochemistry

Enzyme immunohistochemistry was applied on 4 μm sections of formalin-fixed paraffin-embedded healthy conjunctival (*n* = 3) and pterygium (*n* = 7) samples. Prior to staining, all sections were deparaffinized in xylene and rehydrated passing through a series of alcohol solutions in descending concentration. Heat-induced epitope retrieval was carried out in a steamer at 90°C for 30 min in 1 mM EDTA, 10 mM Tris/HCl solution at pH 9.0. After short rinsing with 0.02 M sodium phosphate buffer (PBS) (pH = 7.4), all slides were immersed for 30 min in 0.045% hydrogen peroxide (H_2_O_2_) solution in 0.02 M PBS to quench endogenous peroxidase activity. Slides were rinsed again in 0.02 M PBS. Non-specific binding was blocked for 30 minutes with 5% normal goat serum (NGS) or with 5% normal horse serum (NHS) in 1% skim milk powder and 0.25% gelatin from cold water fish skin (CWFS) added to 0.02 M PBS with 0.1% Triton X-100 at room temperature. Sections were incubated for 1 h at room temperature with primary antibodies against FN1 (1:100, F6140, Sigma-Aldrich, Taufkirchen, Germany) or SPARC (1:200, HPA002989, Sigma-Aldrich, Taufkirchen, Germany). Primary antibodies were diluted in 0.5% bovine serum albumin (BSA), 0.25% CWFS and 1% NGS or 1% NHS, respectively, dissolved in 0.02 M PBS. Negative controls were run by omitting primary antibodies. Following extensive washing with 0.02 M PBS, secondary antibody staining was carried out at room temperature for 30 min with horse anti-mouse biotinylated IgG (1:200, BA-2001, Vector Laboratories, Burlingame, CA, USA) or with goat anti-rabbit biotinylated IgG (1:200, BA-1000, Vector Laboratories, Burlingame, CA, USA) antibodies. Both secondary antibodies were diluted in a solution of 1% NHS or 1% NGS in 0.02 M PBS. Signal amplification was based on avidin-biotin complex (ABC) method (Vectastain^®^ Elite ABC-HRP Kit, PK-6100, Vector Laboratories, Burlingame, CA, USA) followed by 3,3'-diaminobenzidine (DAB) tetrahydrochloride-peroxidase-nickel treatment for visualization and intensification. Finally, sections were counterstained with hematoxylin. Representative images were taken with Jenoptik Progress Gryphax® camera coupled to a Zeiss Axio Imager A1 microscope equipped with a 20x air objective (0.5 NA).

### RNA Isolation

After melting the paraffin block, the pterygium, as well as the control FFPE samples were stored in tubes until RNA isolation, which was performed as previously described (21, 22). Briefly, total RNA was isolated from FFPE samples using the Quick-RNA FFPE Kit (Zymo Research). Following a DNAse I digestion using the Baseline-ZERO kit (Epicentre), the RNA concentration was measured with the Qubit RNA HS Assay Kit on a Qubit Fluorometer (Life Technologies). The RNA quality was determined with the RNA Pico Sensitivity Assay on a LabChip GXII Touch (PerkinElmer).

### RNA Sequencing

RNA sequencing was performed using massive analysis of cDNA ends (MACE), a 3'-RNA sequencing method, as previously described (21, 22). We recently demonstrated that MACE allows sequencing of FFPE samples with high accuracy (17). Briefly, 16 barcoded libraries comprising unique molecule identifiers were sequenced on the NextSeq 500 (Illumina) with 1 × 75 bp. PCR bias was removed using unique molecular identifiers.

### Bioinformatics

Sequencing data (fastq files) were uploaded to and analyzed on the Galaxy web platform (usegalaxy.eu) (23), as previously described (24). Quality control was performed with *FastQC Galaxy Version 0.72* (http://www.bioinformatics.babraham.ac.uk/projects/fastqc/ last access on 06/14/2020). Reads were mapped to the human reference genome (Gencode, release 34, hg38) with *RNA STAR Galaxy Version 2.7.2b* (25) with default parameters using the Gencode annotation file (Gencode, release 34, https://www.gencodegenes.org/human/releases.html). Reads mapped to the human reference genome were counted using *featureCounts Galaxy Version 1.6.4* (26) with default parameters using the aforementioned annotation file. The output of featureCounts was imported to RStudio (Version 1.2.1335, R Version 3.5.3). Gene symbols and gene types were determined based on ENSEMBL release 100 (Human genes, download on 05/25/2020) (27). Genes with zero reads in all samples were removed from analysis. Principal component analysis (PCA) (28) was applied to check for potential batch effects. Differential gene expression was analyzed using the R package DESeq2 Version 1.22.2 (28) with default parameters (Benjamini-Hochberg adjusted *p*-values). Transcripts with log2 fold change (log2 FC) > 2 or < −2 and adjusted *p* < 0.05 were considered as differentially expressed genes (DEG). Heatmaps were created with the R package *ComplexHeatmap 1.20.0* (29). Other data visualization was performed using the *ggplot2* package (30). Gene enrichment analysis and its visualization were done using the R package *clusterProfiler 3.10.1* (31). Cell type enrichment analysis was performed using xCell (32). The tool uses the transcriptomic signatures of 64 distinct immune and stroma cell types to estimate the relative contributions of these cells to a bulk RNA transcriptome. Transcripts per million were calculated as an input for the analysis based on the output of *featureCounts* (assigned reads and feature length), as previously described (33). xCell enrichment scores were compared between different groups using the Mann-Whitney *U*-test.

Pterygia-specific marker genes were determined by calculating DEG between pterygia and healthy conjunctiva as well as conjunctival papilloma, squamous cell carcinoma and melanoma in a first step (training group). The transcriptional profiles of these 3 conjunctival pathologies were recently generated and published by our group using identical sequencing methods (17–19). Only transcripts with log2 FC > 2 and adjusted *p* < 0.001 were considered for further analysis. Subsequently, the Pearson correlation between each gene and diagnosis was calculated. All genes were filtered for Pearson *p* < 0.001 and then arranged by their correlation coefficient. Additionally, the identified pterygia-specific marker genes were validated using transcriptomic data of cultured human pterygium cells from two different studies (13, 14) (validation group). For this purpose, the training as well as the validation group were integrated into a single DESeq2 model to obtain normalized reads of the specific factors previously defined in the training group. Additionally, the 10th and the 75th percentile of expression of each gene in each tissue type were calculated. Only genes for which the 10th percentile of expression in pterygia (training group) was higher than the 75th percentile of expression in all other tissues (training group) were considered as pterygia-specific genes. The validation group was used to determine specificity of the identified marker in two external datasets. The specificity in the validation group was quantified as the difference between the 10th percentile of expression in pterygia (validation group) and the 75th percentile of expression in all other tissues. Among the genes being specific in training and validation groups, the top specific factors were determined based on the difference between the 10th percentile of expression in pterygia (training and validation group) and the 75th percentile of expression in all other tissues (training group).

## Results

### Patient Characteristics

A total of 26 conjunctival samples were included in this study, including 8 pterygium and 8 healthy conjunctival specimens for transcriptome analysis and 7 pterygium and 3 healthy control samples for immunohistochemistry. Basic demographic data are summarized in Table 1. In addition, 26 patients with neoplastic conjunctival lesions, including conjunctival melanoma (*n* = 12), squamous cell carcinoma (SCC) (*n* = 7) and papilloma (*n* = 7), recently published by our group (18, 19), were included to identify pterygium-specific markers. Mean age at surgery was 58.9 (17.9) years for melanoma, 69.6 (13.3) years for SCC and 37.1 (24.3) years for papilloma. There were 3, 5, and 3 male patients in the melanoma, SCC and papilloma group, respectively.

**Table 1:** Demographic data.

	**Pterygia**	**Healthy conjunctiva**	***p***
**RNA-sequencing**			
n	8	8	-
Age at surgery (y)	57.6 (8.5)	55.8 (7.9)	ns
Sex (m/f)	6/2	6/2	ns
**Immunohistochemistry**			
n	7	3	-
Age at surgery (y)	54.6 (7.0)	49.5 (6.5)	ns
Sex (m/f)	5/2	2/1	ns

*Data is shown as mean (standard deviation) or as absolute numbers. Ns, not significant (p > 0.05)*.

### Unsupervised Transcriptomic Analysis

**Figure 1:**
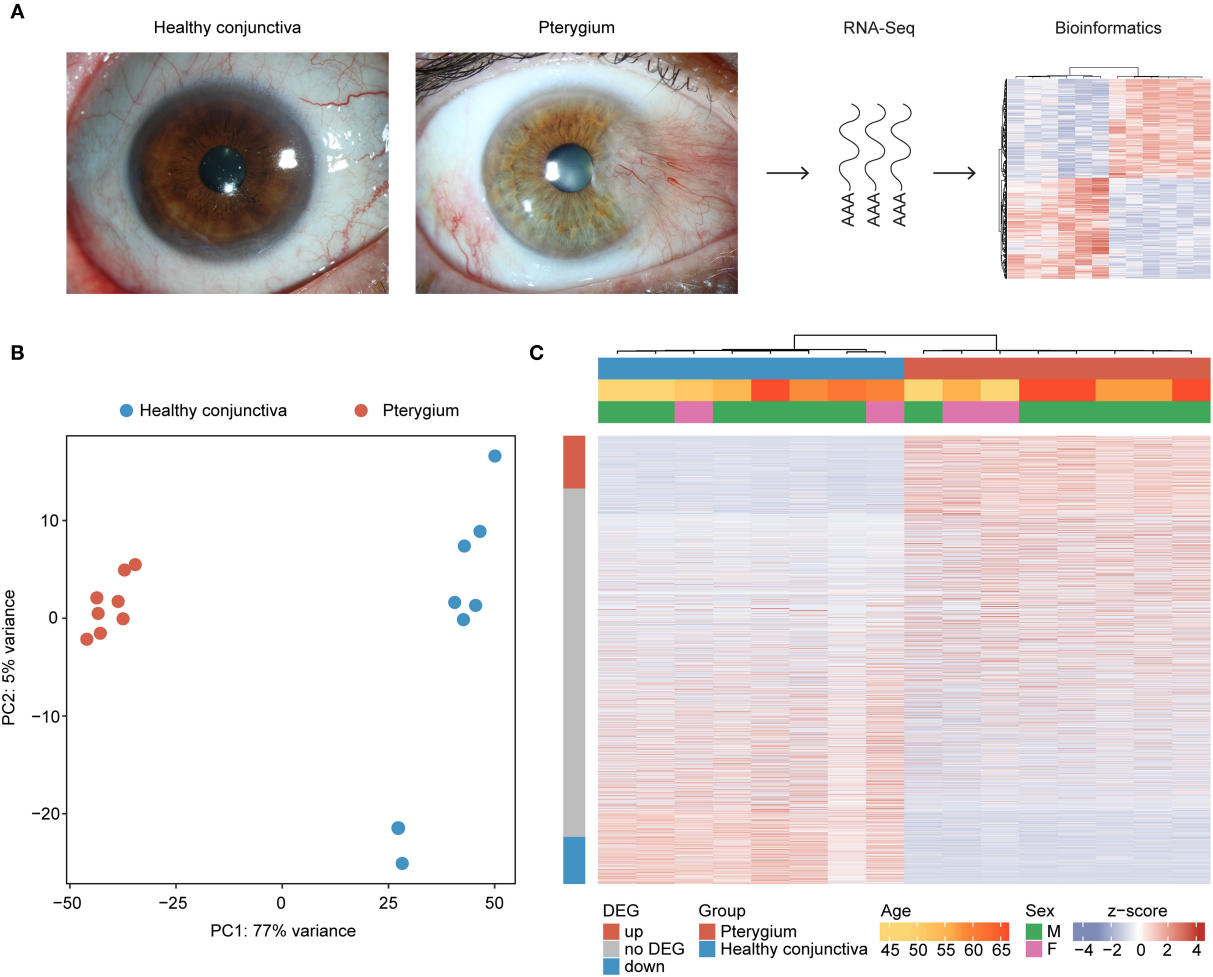
Unsupervised cluster analysis of eight pterygium and eight healthy conjunctival specimens. **(A)** Representative photographs of pterygium and healthy conjunctiva as well as an overview of the study design. **(B)** Principal component analysis. **(C)** Heatmap visualizing the expression of 15.984 genes with at least one read in eight pterygium and eight healthy conjunctival specimens. Basic demographic and clinical data are shown above. Each column represents one sample (red: pterygium, blue: healthy conjunctiva) and each row one expressed gene. The rows are ordered according to the log2 fold change between both groups, placing the upregulated genes in pterygia at the top (see row annotation red) and the downregulated genes at the bottom of the heatmap (see row annotation blue). Unsupervised clustering was performed for the columns (see dendrogram). The z-score represents a gene's expression in relation to its mean expression by standard deviation units (red: upregulation, blue: downregulation). DEG, differentially expressed gene.

The transcriptional profile of 8 pterygium and 8 healthy conjunctival specimens was analyzed using MACE RNA sequencing (Figure 1A). Unsupervised analysis revealed distinct differences in the transcriptome of pterygium samples when compared to healthy conjunctiva (Figures 1B,C). No obvious differences in transcriptional profile depending on age or sex could be identified (Figure 1C).

### Cellular Microenvironment of Pterygia

**Figure 2:**
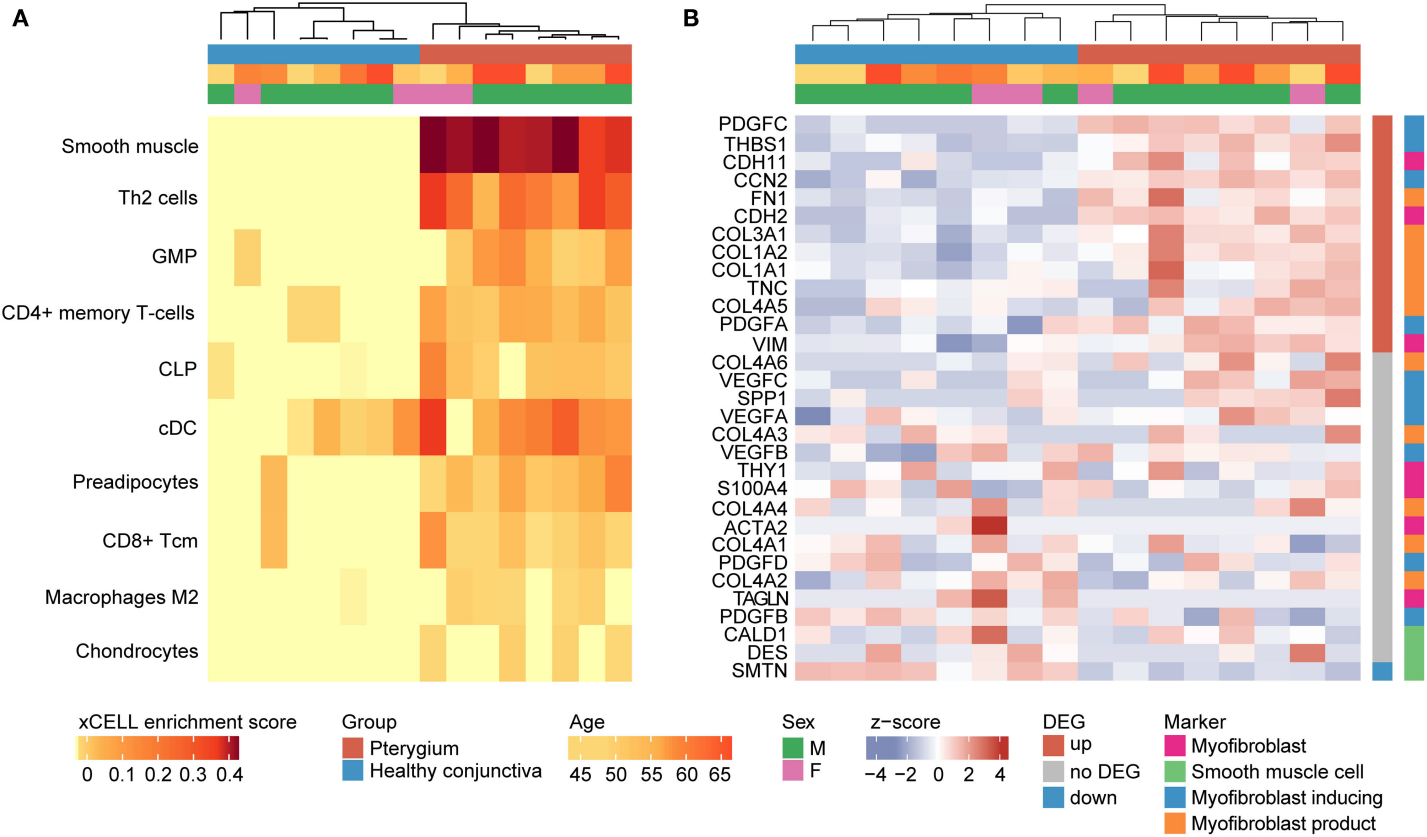
Cellular microenvironment of pterygia **(A)** cell type enrichment analysis using xCell. The tool uses gene expression profiles of 64 immune and stromal cell types to calculate cell type enrichment scores. Heatmap illustrating xCell enrichment scores of 10 of the 64 cell types which were significantly enriched in pterygia compared to healthy conjunctiva (*p* < 0.05, Mann-Whitney *U*-test). Each row represents one cell type, each column represents one sample. Rows are ordered according to the fold change of mean enrichment scores. Columns are clustered according to similarities in xCell enrichment scores (see dendrogram). Basic demographic and clinical data are shown above. Th2, type 2 T-helper cells; GMP, granulocyte-monocyte progenitor; CLP, common lymphoid progenitor; cDC, classical DC; Tcm, central memory T cell. **(B)** Since according to **(A)** smooth muscle cells seemed to be the dominant cell type in pterygia, the expression of known markers of smooth muscle cells as well as of myofibroblasts was subsequently analyzed **(B)** revealing a myofibroblast-specific expression profile.

Cell type enrichment analysis using xCell (32) revealed that in pterygium samples, the marker genes of different stroma and immune cell types were detected more frequently than in healthy conjunctiva, among them, most notably smooth muscle cells, type 2 T-helper (Th2) cells and granulocyte-monocyte progenitor (GMP) cells. In addition, pterygium samples were characterized by the enrichment of CD4+ memory T–cells, common lymphoid progenitor (CLP) cells, classical dendritic cells (cDC), preadipocytes, CD8+ T-cells, M2 macrophages and chondrocytes (Figure 2A). The cell type analysis also revealed that pterygium and control samples clustered according to their histological diagnosis based on their cell type enrichment scores, indicating a significant modification of the cellular microenvironment in pterygia (Figure 2A). This finding was unaffected when all 64 cell types were included in the analysis (data not shown). According to the results of the xCell analysis, smooth muscle cells seemed to be the dominant cell type in pterygia. Since myofibroblast-specific gene signatures are not included in the xCell algorithm, the expression of known markers of smooth muscle cells as well as of myofibroblasts (34, 35) was subsequently analyzed (Figure 2B). Myofibroblast-specific genes were found to be either significantly upregulated in pterygia or expressed at similar levels between both groups. Interestingly, none of the marker genes was significantly downregulated in pterygium specimens. In addition, analyzing the expression of *CALD1, DES*, and *SMTN*, which are three known markers of smooth muscle cells being absent in myofibroblasts (34), revealed a significant downregulation or comparable expression in pterygium samples when compared to healthy conjunctiva. In summary, these results indicate myofibroblasts to be the most differentially enriched cell type in pterygium specimens.

### Transcriptional Characterization of Pterygia

**Figure 3:**
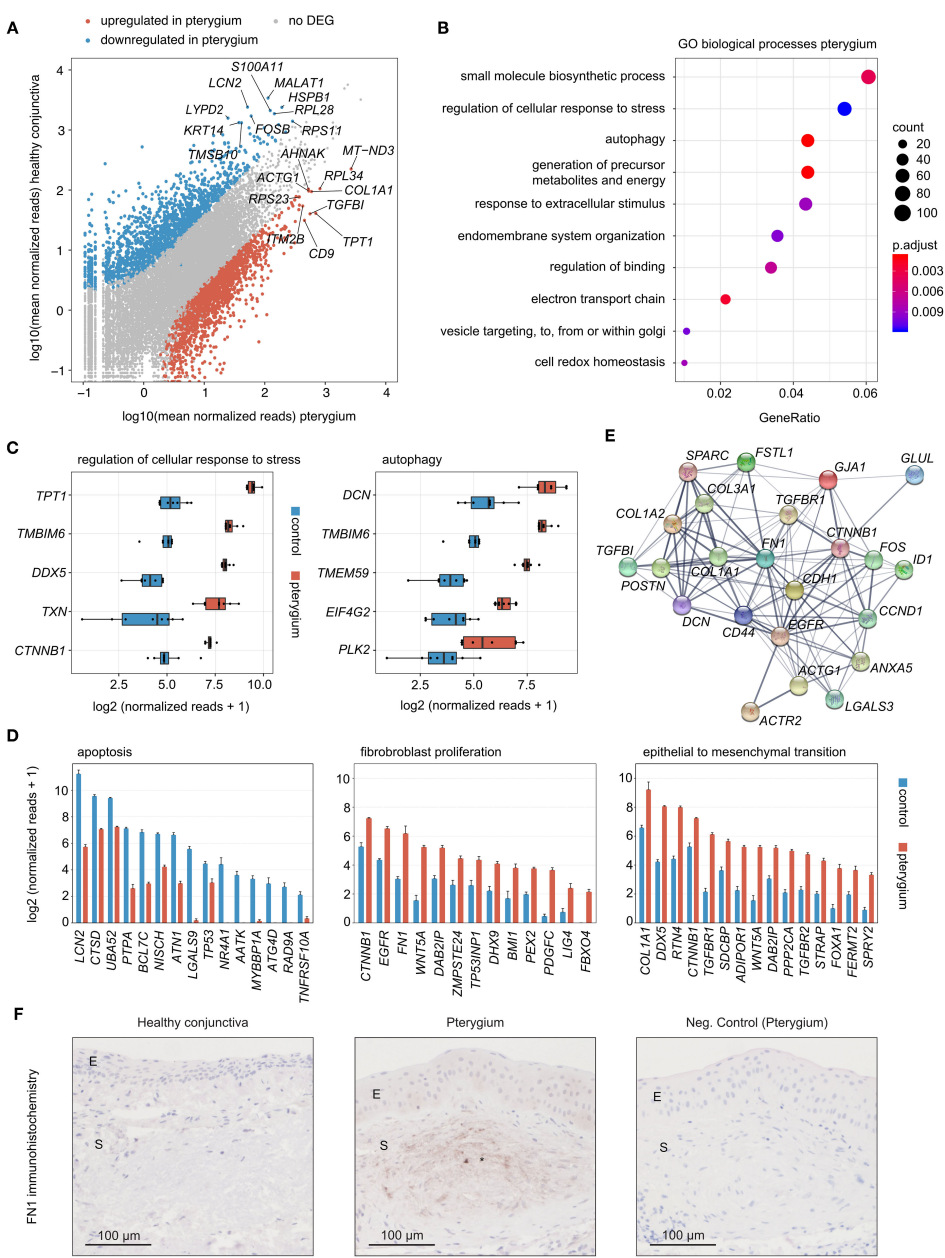
**(A)** Read plot visualizing DEG in pterygia and healthy conjunctiva. The top 10 highest expressed DEG of both groups are labeled. **(B)** Gene ontology (GO) analysis of the upregulated genes in pterygia. The top ten biological processes, which the DEG were involved in, are shown in the dot plot. The size of the dots represents the number of associated genes (count). The adjusted *p*-value of each GO term is shown by color. The gene ratio describes the ratio of the count to the number of all DEG. **(C)** Box plots illustrating normalized reads of the top five expressed factors of two disease-relevant GO terms. **(D)** Bar plots visualizing expression of top 15 downregulated DEG associated to apoptosis (GO:0006915), as well as upregulated DEG associated to fibroblast proliferation (GO:0048144) and epithelial to mesenchymal transition (GO:0001837) (visualized as mean + standard error of means). **(C,D)** Benjamini-Hochberg adjusted *p*-values were smaller than 0.001 for each gene, with the exception of *ATG4D, ZMPSTE24, TP53*, and *NR4A1* with an adjusted *p*-value below 0.01 and *BMI1, FERMT2, TNFRSF10A, TP53INP1*, and *LIG4* with an adjusted *p*-value below 0.05. **(E)** STRING network of the key pterygia-associated factors selected according to the number of connections. **(F)** Immunohistochemistry of FN1 in pterygia and healthy conjunctiva. E, epithelium; S, stroma; asterisk, stromal immunoreactivity against FN1.

Differential gene expression analysis revealed 1881 up- and 1676 downregulated genes in pterygia compared to healthy conjunctiva (Figure 3A). Among all upregulated genes, *MT-ND3* (Mitochondrially Encoded NADH:Ubiquinone Oxidoreductase Core Subunit 3), *RPL34* (Ribosomal Protein L34), *TPT1* (Tumor Protein, Translationally-Controlled 1), *COL1A1* (Collagen Type I Alpha 1 Chain) and *TGFBI* (Transforming Growth Factor Beta Induced) were the top 5 expressed genes (Figure 3A). Gene ontology (GO) analysis revealed, that the upregulated genes were most significantly involved in biological processes such as regulation of cellular response to stress, autophagy, response to extracellular stimulus, electron transport chain and cell redox homeostasis (Figure 3B). The top five expressed genes in pterygia associated to regulation of cellular response to stress were *TPT1, TMBIM6* (Transmembrane BAX Inhibitor Motif Containing 6)*, DDX5* (DEAD-Box Helicase 5)*, TXN* (Thioredoxin) and *CTNNB1* (Catenin Beta 1) (Figure 3C). *DCN* (Decorin), *TMBIM6, TMEM59* (Transmembrane Protein 59), *EIF4G2* (Eukaryotic Translation Initiation Factor 4 Gamma 2) and *PLK2* (Polo Like Kinase 2) were the top 5 expressed genes in autophagy (Figure 3C). When looking at the disease-relevant biological processes fibroblast proliferation (GO:0048144) and epithelial to mesenchymal transition (GO:0001837), *CTNNB1, COL1A1* and *DDX5*, as well as *CD9* (CD9 Molecule), *RTN4* (Reticulon 4), *LGALS3* (Galectin 3), *EGFR* (Epidermal Growth Factor Receptor), *FN1* (Fibronectin 1), *BMI1* (BMI1 Proto-Oncogene, Polycomb Ring Finger), *PDGFC* (Platelet Derived Growth Factor C), *TGFBR1* (Transforming Growth Factor Beta Receptor 1) and *-2* appeared among the top expressed genes as well (Figure 3D). In addition, several factors involved in apoptosis were found to be significantly downregulated in pterygia, among them Lipocalin 2 (*LCN2*), Cathepsin D (*CTSD*), Nischarin (*NISCH*), MYB Binding Protein 1a (*MYBBP1A*) and TNF Receptor Superfamily Member 10a (*TNFRSF10A*) (Figure 3D). Among the upregulated genes in pterygia, a STRING analysis (36) identified *FN1* as a key disease-associated factor (Figure 3E). We therefore analyzed the protein expression of FN1 in pterygia and healthy controls applying immunohistochemistry (Figure 3F). These experiments revealed a significant stromal immunoreactivity against FN1 in 4 out of 7 pterygia, which was absent in controls, as well as a slightly increased epithelial staining in one pterygium.

### Pterygia-Specific Marker Genes

**Figure 4:**
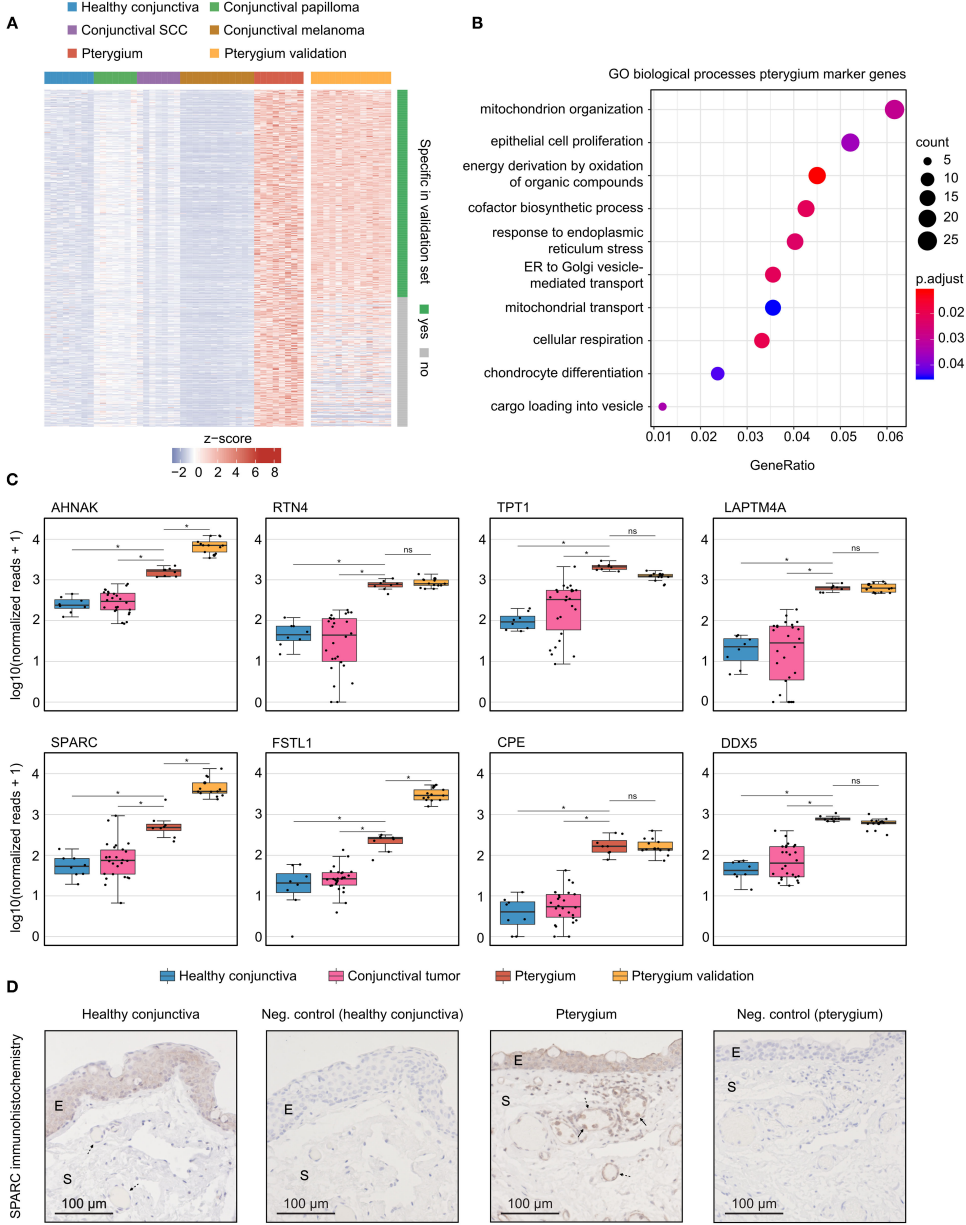
Pterygia-specific marker genes. **(A)** Heatmap visualizing 731 identified marker genes in pterygia when compared to healthy conjunctiva, conjunctival papilloma, conjunctival squamous cell carcinoma and conjunctival melanoma. External validation was performed by including transcriptomic data of cultured human pterygium cells from two different studies (13, 14) (see orange columns). Each column represents one sample and each row represents one gene (see colored legend for different tissue types). Rows are ordered according to specificity in the validation datasets. Of a total of 731 identified marker genes, 450 were likewise specific in the validation dataset (see row annotation and methods for details). The z-score represents a gene's expression in relation to its mean expression by standard deviation units (red: upregulation, blue: downregulation). **(B)** Gene ontology (GO) analysis of the pterygia marker genes [green rows in **(A)**]. The top 10 biological processes, which the marker genes were involved in, are shown in the dot plot. The size of the dots represents the number of associated genes (count). The adjusted *p*-value of each GO term is shown by color. The gene ratio describes the ratio of the count to the number of all marker genes. **(C)** Boxplots illustrating expression of the 4 most specific pterygia marker genes (first row) as well as 4 selected markers (from top 40, second row). *, adjusted *p* < 0.001; ns, not significant. **(D)** Immunohistochemical validation of SPARC protein expression in pterygia and controls. E, epithelium; S, stroma; dashed arrow, vessel; solid arrow, mononuclear cell.

Pterygia-specific marker genes were further specified by determining DEG between pterygia and healthy conjunctiva as well as conjunctival papilloma, squamous cell carcinoma and melanoma in a first step, followed by calculating the correlation coefficient between each gene and diagnosis in a second step. All DEG were filtered for Pearson *p* < 0.001 and then arranged by their correlation coefficient. The identified marker genes were validated using transcriptomic data of cultured human pterygium cells from two different studies (13, 14) (see Methods for details). The expression profile of these genes is visualized in the heatmap in Figure 4A. Of the 731 identified genes, 450 were also specific for pterygia in the validation data (Figure 4A and Supplementary Table 1). GO analysis revealed that these marker genes were mainly involved in biological processes such as mitochondrial organization, epithelial cell proliferation, response to endoplasmic reticulum stress, cellular respiration and chondrocyte differentiation (Figure 4B). The most specific pterygia markers identified by integrating the samples sequenced in the current study and the validation samples from the literature (13, 14) are illustrated in the boxplots in Figure 4C. Genes including *RTN4, TPT1, DDX5, AHNAK* (AHNAK Nucleoprotein), *FSTL1* (Follistatin Like 1) and *SPARC* were identified as pterygia-specific marker genes reaching high classification accuracy in our as well as in external validation data (Figure 4C). Since *SPARC* was one of the most specific pterygia marker genes, we analyzed the protein expression of SPARC in pterygia and healthy controls by means of immunohistochemistry (Figure 4D). These experiments revealed a distinct epithelial and stromal immunoreactivity against SPARC in 4 and 3 out of 4 pterygia, respectively, whereas a similar epithelial and absent stromal reactivity was detected in 3 controls. Interestingly, the majority of vessels in pterygia exhibited significant immunoreactivity against SPARC, whereas the vasculature in controls was SPARC-negative in most cases. In addition, there were significantly higher numbers of intra- and perivascular mononuclear cells in pterygia than in controls, which were SPARC-positive and adjacent to the vessel wall more frequently (Figure 4D).

## Discussion

Gene expression analysis can provide important insights into the molecular mechanisms of a disease and has helped to define new therapeutic targets in a variety of pathologies. The present study applies RNA sequencing to gain detailed insights into the underlying molecular mechanisms and, for the first time, uses bioinformatic cell type deconvolution analysis to decipher the cellular microenvironment of pterygia. In addition, this study identifies a variety of new pterygia-specific markers by including not only healthy conjunctiva, but also various ocular surface tumors as controls and validates these markers by independent and published transcriptome datasets as well as by immunohistochemistry.

So far, there are only a limited number of studies that have applied RNA sequencing on pterygia, including two studies using cultured pterygium cells (13, 14) and two recently published studies based on surgically removed pterygium tissue (15, 16). However, these studies are limited by small samples sizes and by controls obtained from pterygia-affected eyes (15), so an influence of the disease as well as associated environmental factors, such as ultraviolet radiation, on control tissue cannot be excluded. In addition, the studies are limited by the use of postmortem control tissue (16), in which significant RNA degradation occurs during the time from death to conservation (37, 38). To circumvent these limitations, the present study uses tissue specimens which were formalin-fixed (FFPE) immediately after surgical excision as well as control tissue which was excised from patients with healthy ocular surfaces. Since in FFPE samples, RNA is exposed to chemical degradation primarily at the 5' end (39), the 3' RNA sequencing method Massive Analysis of cDNA Ends (MACE) was applied, which allows sequencing of FFPE samples with high accuracy (22).

Transcriptome-based cell type enrichment analysis using xCell (32) revealed that the cellular microenvironment of pterygia was predominantly characterized by an enrichment of smooth muscle cells, as well as numerous immune cell types, including type 2 T-helper cells, CD4+ memory T–cells, classical dendritic cells, CD8+ T-cells and M2 macrophages. Since smooth muscle cells seemed to be the most significantly enriched cell type in pterygia, the expression of known markers of smooth muscle cells as well as of myofibroblasts (34, 35) was analyzed, the latter being not included within the xCell algorithm (Figure 2B). The results indicated that myofibroblasts represent the most differentially enriched cell type in pterygium specimens, which supports their assumed role in the pathogenesis of the disease (2, 35, 40–42). Current evidence suggests that myofibroblasts emerge from conjunctival epithelial cells through the process of epithelial-mesenchymal transition (3, 35). The significance of this process is supported by the data of this study showing an upregulation of different mesenchymal markers in pterygium samples, such as *VIM* (15), *CDH2* and *S100A4* (3, 35). Looking at immune cell types, pterygium samples were predominantly characterized by the enrichment of T cells. This finding is in line with previous studies, revealing T-lymphocytes to be the dominant immune cell type in pterygia (43–45), with increased levels of both CD4- and CD8-positive T-cells (44, 45). In addition, antigen-presenting cells, among them in particular dendritic cells and macrophages, have been shown to be increased in pterygia (46). These findings validate the results of the xCell analysis on the one hand and indicate immunological mechanisms being involved in the pathophysiology of the disease on the other hand that could represent potential therapeutic targets.

The transcriptional profile of pterygium specimens provided in this study differed significantly from healthy conjunctival samples and revealed 1881 up- and 1676 downregulated genes. Gene ontology (GO) analysis demonstrated, that these genes were mainly involved in biological processes such as autophagy and regulation of cellular response to stress, the latter thought to play a role in epithelial-mesenchymal transition (47). The most highly expressed gene among the autophagy-associated genes was Decorin, which is known to induce autophagy and concurrently to decrease apoptosis (48). It is interesting to note that in addition, a variety of pro-apoptotic factors were found to be significantly downregulated in pterygia when compared to control specimens, among them *LCN2* (49), *CTSD* (50), *NISCH* (51), and *MYBBP1A* (52). These results are in line with microarray studies by He et al. who reported that 36% of genes downregulated in pterygia were involved in apoptosis (53). In particular, a *LCN2* knockdown is associated with a decreased activation of the mitochondrial apoptosis pathway (49) underscoring its pro-apoptotic function. *CTSD* was found to promote apoptosis by activating *CASP3* (50). Downregulation of *NISCH* has recently been reported to reduce oxidative stress-induced apoptosis (51), while *MYBBP1A* decreased breast cancer tumorigenesis by activating *TP53* (52). In line with these results, we also found a significant reduction of *TP53* and *TNFRSF10A* in pterygia, which are important regulators of apoptosis (54, 55). These findings may explain the reported low number of apoptotic cells in pterygia compared to normal conjunctiva (56) and emphasize that pterygia might develop as a consequence of an interruption of the normal apoptosis process in the conjunctiva (56). Among the genes involved in regulation of cellular response to stress, *TPT1* was the top expressed DEG. The microRNA *miR-455-3p* has been shown to repress cell proliferation in colorectal cancer cells by targeting *TPT1* (57), therefore representing a new potential therapeutic target for pterygia. *DDX5*, also among the top expressed DEG, is known to play a role in tumor cell proliferation and epithelial-mesenchymal transition in different malignancies (58–61) and may therefore also represent a therapeutic approach for the treatment of pterygia. Furthermore, this study identifies *FN1* as one of the key pterygia-associated factors on the RNA level, which was validated by immunohistochemistry revealing significant stromal immunoreactivity against FN1 in pterygia which was absent in controls, a finding which is in accordance with previously published results (8, 10, 15, 16). FN1 is a glycoprotein of the extracellular matrix being involved in wound healing, cell adhesion, proliferation and migration (62). Knockdown of *FN1* resulted in reduced cell proliferation and migration in colorectal cancer cells (62), suggesting *FN1* as a potential therapeutic target for pterygia.

In search of pterygium-specific marker genes, this study compared the transcriptional profile of pterygia to healthy conjunctiva, as well as to different tumors of the ocular surface, such as conjunctival squamous cell carcinoma, papilloma and melanoma. In addition, the results were validated using transcriptomic data of cultured human pterygium cells from two different studies (13, 14). Following this approach, this study identified 450 marker genes, which were also specific for pterygia in the validation data. *SPARC* was identified as one of the most specific marker genes on the RNA level, a finding which was validated on the protein level by means of immunohistochemistry. Apart from a significant epithelial immunoreactivity in pterygia, we observed a distinct staining of most of the vessels in pterygia, whereas the vasculature was predominantly SPARC-negative in controls. In addition, there were higher numbers of intra- and perivascular mononuclear cells in pterygia than in controls, which were SPARC-positive and adjacent to the vessel wall more frequently. *SPARC* is known to regulate the interactions between cells and their extracellular matrix, thereby modulating cell adhesion, proliferation and differentiation in diseases such as gastric cancer (63), lung cancer, idiopathic pulmonary fibrosis (64) as well as in pterygia (65). *In vitro* studies using endothelial cells have shown increased SPARC expression in proliferating endothelial cells with elevated expression in a proinflammatory milieu (66). In addition, leukocyte-derived SPARC is involved in transendothelial leukocyte migration by interacting with the endothelial cell surface molecule VCAM-1 (vascular cell adhesion molecule 1), which results in enhanced transendothelial permeability by recombinant SPARC (66) and reduced leukocyte recruitment in *SPARC* knockout mice (67). These results suggest that increased SPARC expression in vessels may be related to proliferating endothelial cells—-known as a pterygia-associated process (15)—-and in transendothelial immune cell migration in the pro-inflammatory milieu in pterygia. Further studies are needed to identify the exact cell type of SPARC-positive peri- and intravascular mononuclear cells and to investigate their pathophysiological involvement in pterygia.

While some of the aforementioned genes have been previously discussed in the context of pterygia, this study identifies several pterygia-specific factors that have not previously been associated with the disease, such as *AHNAK, RTN4, TPT1*, and *FSTL1*. *AHNAK* is involved in epithelial mesenchymal transition in response to Transforming Growth Factor beta thereby promoting tumor metastasis (68). RNA interference-mediated knockdown of *RTN4* has been shown to inhibit cell growth of human colorectal cancer cells (69). Likewise, knockdown of *FSTL1* has been reported to inhibit cell proliferation and migration of colorectal cancer cells (70), thus representing a potential therapeutic target for pterygia. Further studies are necessary to investigate the involvement of the presented factors and signaling pathways in the development of pterygia in more detail and to validate them as potential therapeutic targets for the treatment of the disease.

The results of this study provide new translational implications for potential new therapeutic avenues for this common ocular surface disease. In general, therapeutic modulation of epithelial-mesenchymal transition, apoptosis, and autophagy, which all were identified as key pterygium-associated processes, as well as immunomodulation with special emphasis on T lymphocytes, may represent a potential therapeutic strategy. More specifically, therapeutic modulation of *FN1, TPT1, RTN4*, and *FSTL1*, for which knockdown has been shown to result in reduced cell proliferation and migration in cancer cells (57, 62, 69, 70), may represent new potential therapeutic avenues for pterygia as well. In addition, the results of this study indicate that increased SPARC expression in vessels and mononuclear immune cells may be involved in transendothelial immune cell migration in the pro-inflammatory milieu in pterygia. Interestingly, a recently published study (71) demonstrated that silencing of *SPARC* inhibited the expression of profibrotic markers, such as alpha smooth muscle actin and *FN1* in human pterygium fibroblasts and also mitigated their migration and contractile phenotype. These results strongly suggest, that *SPARC* may be a promising therapeutic target for the treatment of pterygia.

We acknowledge that this study is limited by its retrospective single center design. Furthermore, in contrast to single cell RNA sequencing (scRNA), bulk RNA sequencing cannot provide insights into cell heterogeneity and thus cannot reveal cell-specific transcriptional profiles to identify possible subtypes of cells. However, scRNA sequencing is not feasible on FFPE samples. Therefore, we employed a bulk RNA sequencing-based cell type enrichment analysis using xCell (32), which is one of the most accurate tools available (72), in combination with known cell type marker genes (34, 35), to characterize the cell types involved in the microenvironment of pterygia. It is important to note that these results are based on *in silico* analysis and have not been validated histologically in the present study. However, some of the results recapitulate the findings of previous studies by other groups, supporting the results of the xCell analysis (2, 35, 43–46).

In summary, the present study provides new insights into the cellular microenvironment and the transcriptional profile of pterygia and applies immunohistochemistry to validate key pterygia-associated factors. The results of this study contribute to an improved understanding of the pathophysiological processes underlying the disease and reveal new diagnostic biomarkers that may enable new options of targeted therapy for pterygia.

## Data Availability Statement

The sequencing data are available in the Gene Expression Omnibus Database under the following accession numbers: GSE155776 (pterygia, healthy conjunctiva), GSE148387 (healthy conjunctiva and conjunctival melanoma) and GSE149004 (healthy conjunctiva, conjunctival carcinoma and papilloma). Sequencing data of cultured human pterygium cells (13, 14) published under the accession numbers GSE34736 and GSE58441 were used for validation.

## Ethics Statement

Ethics approval was granted from local Ethics Committees. The patients provided their written informed consent to participate in this study.

## Author Contributions

JW and CL designed the study, collected, analyzed, and interpreted the data. RH pre-processed tissue and performed immunohistochemistry. Ophthalmopathologic diagnoses were made by CA-H. JW performed bioinformatic analyses, generated figures, carried out literature research, and wrote the original draft of the manuscript. All authors were involved in reviewing and editing the paper and had final approval of the submitted and published version.

## Conflict of Interest

The authors declare that the research was conducted in the absence of any commercial or financial relationships that could be construed as a potential conflict of interest.

## Publisher's Note

All claims expressed in this article are solely those of the authors and do not necessarily represent those of their affiliated organizations, or those of the publisher, the editors and the reviewers. Any product that may be evaluated in this article, or claim that may be made by its manufacturer, is not guaranteed or endorsed by the publisher.
